# Supplementary material for: Comparative genomics provides new insights into the diversity, physiology, and sexuality of the only industrially exploited tremellomycete: Phaffia rhodozyma
Source: BMC Genomics. 2016 Nov 9;17:901. doi: 10.1186/s12864-016-3244-7 (PMC5103461; doi:10.1186/s12864-016-3244-7)
Supplement: Additional file 6: — List of orphan genes with links to PFAM (related to Additional file 1: Table S1). (ZIP 1428 kb) [file 12864_2016_3244_MOESM6_ESM.zip › PFAM_UNIQUE/G03566_P.html]

#                                                                            --- full sequence --- -------------- this domain -------------   hmm coord   ali coord   env coord
  
# target name        accession   tlen query name           accession   qlen   E-value  score  bias   #  of  c-Evalue  i-Evalue  score  bias  from    to  from    to  from    to  acc description of target
  
#------------------- ---------- ----- -------------------- ---------- ----- --------- ------ ----- --- --- --------- --------- ------ ----- ----- ----- ----- ----- ----- ----- ---- ---------------------
  
G03566\_P             -             92 DUF1651              PF07864.6     75     0.086   11.6   0.5   1   1     2e-05      0.12   11.2   0.5    21    68    40    87    25    89 0.85 - Protein of unknown function (DUF1651)  
G03566\_P             -             92 DUF4164              PF13747.1     89     0.082   11.7   1.3   1   2         6   4.5e+03   -3.4   0.0    74    79     7    12     3    19 0.41 - Domain of unknown function (DUF4164)  
G03566\_P             -             92 DUF4164              PF13747.1     89     0.082   11.7   1.3   2   2   0.00017      0.12   11.2   1.0    31    67    48    83    39    85 0.86 - Domain of unknown function (DUF4164)  
G03566\_P             -             92 HSBP1                PF06825.7     54     0.037   12.3   0.6   1   1   1.9e-05     0.056   11.7   0.6    26    42    67    83    53    89 0.84 - Heat shock factor binding protein 1  
G03566\_P             -             92 Matrilin\_ccoil       PF10393.4     47       0.1   10.7   2.2   1   1   4.9e-05      0.15   10.2   2.2    18    44    58    83    54    85 0.93 - Trimeric coiled-coil oligomerisation domain of matrilin  
G03566\_P             -             92 T2SM                 PF04612.7    160       0.2   10.1   2.2   1   1   8.3e-05      0.25    9.8   2.2    42    74    53    85    49    91 0.89 - Type II secretion system (T2SS), protein M  
G03566\_P             -             92 Tup\_N                PF08581.5     79     0.055   12.4   0.9   1   1   5.2e-05     0.078   11.9   0.9    20    60    49    90    43    91 0.74 - Tup N-terminal  
G03566\_P             -             92 U1snRNP70\_N          PF12220.3     94       5.4    6.1   7.2   1   2    0.0013       1.1    8.3   0.5    10    33    27    49    21    55 0.84 - U1 small nuclear ribonucleoprotein of 70kDa MW N terminal  
G03566\_P             -             92 U1snRNP70\_N          PF12220.3     94       5.4    6.1   7.2   2   2      0.17   1.4e+02    1.5   2.6    59    85    49    75    44    84 0.63 - U1 small nuclear ribonucleoprotein of 70kDa MW N terminal
